# A Novel Polyvinylpyrrolidone-Stabilized Illite Microparticle with Enhanced Antioxidant and Antibacterial Effect

**DOI:** 10.3390/polym13244275

**Published:** 2021-12-07

**Authors:** Hyeryeon Oh, Jin Sil Lee, Hye Sun Lee, Daekyung Sung, Won Il Choi

**Affiliations:** 1 Center for Convergence Bioceramic Materials, Convergence R&D Division, Korea Institute of Ceramic Engineering and Technology, 202, Osongsaengmyeong 1-ro, Osong-eup, Heungdeok-gu, Cheongju 28160, Korea; hyeryeon.oh@kicet.re.kr (H.O.); jslee92@kicet.re.kr (J.S.L.); hslee@kicet.re.kr (H.S.L.); 2 School of Materials Science and Engineering, Gwangju Institute of Science and Technology, 261 Cheomdan-gwagiro, Buk-gu, Gwangju 500-712, Korea

**Keywords:** illite, polyvinylpyrrolidone, organoclay, antioxidant, antibacterial effect

## Abstract

Illite is a clay mineral that shows antioxidant and antibacterial activities because of the abundance of important clay elements in its structure. However, illite has low bioactivity due to its low solubility and electron-donating ability in aqueous solutions. Therefore, we aimed to develop polyvinylpyrrolidone (PVP)-stabilized illite microparticles (P-lite MPs) via polymer adsorption on illite surfaces. An increasing amount of PVP was used to coat a fixed amount of illite to prepare P-lite MPs of different hydrodynamic diameters in the range of 4–9 μm. These sizes were maintained for 2 weeks during storage in a biological buffer without any noticeable changes. The stabilization of illite microparticles using a hydrophilic PVP polymer improved their aqueous dispersity and free radical-scavenging activity. Since the large surface area of microparticles provides several sites for interactions, the smallest P-lite MP exhibited the highest antioxidant and antibacterial activities. More importantly, the MPs showed effective free radical-scavenging activity in vitro without any cytotoxicity. Therefore, P-lite MPs with improved bioavailability may represent a suitable bioactive material for various industrial and biomedical applications.

## 1. Introduction

Clay minerals are natural aluminosilicates widely deposited on the earth’s surface [1]. They support plant growth as a major component of soils and are traditionally used in pottery, absorbents, and paints [2,3]. Furthermore, they are used in a wide range of applications, including cosmetics, biocides, and pharmaceuticals, because of their characteristic structures and chemical compositions [4,5]. They have been used for treating gastrointestinal and topical diseases since ancient times [6]. The exact mechanism underlying their therapeutic effects remains unknown; however, several studies have evaluated the important factors that affect the anti-inflammatory and antimicrobial activities of clay minerals, including metal element composition, toxin adsorption, oxidation state, pH, and surface properties [7,8]. Thus, the application of clay minerals has attracted attention in the biomedical field for protection against infection and inflammation.

Clay minerals can be classified into five types: kaolinite, illite, chlorite, smectite, and vermiculite [9,10,11]. In particular, illite is known for its bioactivity, large reserves, and potential economic benefits. However, it has low bioavailability in aqueous solutions. Clay minerals exhibit different swelling properties in the presence of water. Smectite has a high cation-exchange capacity and expansion capability, whereas illite is a non-expanding clay [12] because the interlayer cations in illites prevent the entry of water molecules into their structures [13]. Since the antibacterial activity of clay minerals depends on their hydration and release of soluble metal ions, the flocculation of clay minerals has been resolved via polymer adsorption [14,15].

Hydrophilic polymers, such as polystyrene, poly(vinyl alcohol), poly(ethylene glycol), poly(acrylic acid), and poly(methyl methacrylate) have been used as template materials for developing clay minerals [16,17]. In particular, the polymer composites of clay minerals synergistically enhance the bioactivity and cellular uptake in biomedical applications [18]. However, to the best of our knowledge, polyvinylpyrrolidone (PVP) has not yet been used to prepare a composite of illite for enhancing bioactivity and stability. Hydrophilic and biodegradable PVP, a U.S. Food and Drug Administration-approved material, is one of the most useful polymers in technological and pharmaceutical applications [19]. As reported previously, a PVP composite of silica shows good dispersity in an aqueous solution owing to strong hydrogen bonds with each other [20]. Furthermore, the intercalation of PVP into the clay structure has been performed to estimate the surface area of clay minerals [21]. Thus, the stabilization of illite via coating with a PVP polymer may be expected to enable the interactions of illite metal elements in an aqueous solution for enhanced antioxidant and antibacterial activities.

Therefore, in this study, a PVP-stabilized illite microparticle (P-lite MP) was developed to enhance the bioavailability and bioactivity of illite via a simple metal intercalation method between PVP and illite. Different amounts of PVP were used as stabilizing agents, and the resulting microparticles (MPs) were characterized using dynamic light scattering (DLS). The hydrodynamic diameters and size distributions of the MPs were analyzed based on the weight ratio of illite to PVP. Scanning electron microscopy (SEM), Fourier-transform infrared spectroscopy (FTIR), and X-ray photoelectron spectroscopy (XPS) were performed to further determine their physicochemical properties. A colony-counting method was used to evaluate the antibacterial activities of P-lite MPs. Both in situ and in vitro free radical-scavenging assays were carried out to analyze the antioxidant activities of MPs.

## 2. Materials and Methods

### 2.1. Materials

Illite was purchased from Medexx (Seongnam, Korea). PVP (average molecular weight = 10 kDa), ethylenediaminetetraacetic acid (EDTA), phosphate-buffered solution (1 M, pH 7.4), 2-thiobarbituric acid (TBA), trichloroacetic acid (TCA), and 2,2-diphenyl-1-picryhydrazyl (DPPH) were obtained from Sigma-Aldrich (St. Louis, MO, USA). Hydrogen peroxide (H_2_O_2_, 30%) was purchased from Junsei Chemical Co. (Tokyo, Japan). L-Ascorbic acid was purchased from TCI (Tokyo, Japan). Iron (III), chloride (FeCl_3_), and 2-deoxy-D-ribose (99%) were obtained from Alfa Aesar (Ward Hill, MA, USA). Methanol was purchased from SK Chemical (Seongnam, Korea). Deionized (DI) water and phosphate-buffered saline (PBS) were obtained from Hyclone (Logan, UT, USA). The gram-positive bacterium, *Staphylococcus aureus* ATCC 6538, was purchased from the American Type Culture Collection (ATCC; Manassas, VA, USA) for the antibacterial assay. NIH 3T3 mouse embryonic fibroblasts (Korean Cell Line Bank, Seoul, Korea) were cultured in Dulbecco’s Modified Eagle Medium (DMEM; Gibco, Grand Island, NY, USA) supplemented with fetal bovine serum (FBS) from Gibco and antibiotic-antimycotic (AA) from Thermo Fisher Scientific (Waltham, MA, USA). 2′,7′-dichlorodihydrofluorescein diacetate (H2DCFDA) was purchased from Invitrogen (Carlsbad, CA, USA) to analyze the in vitro ROS level.

### 2.2. Preparation of P-Lite MPs

For preparing P-lite MPs, PVP was first dissolved at 25 °C in 5 mL of DI water into different concentrations (50, 100, and 200 mg/mL). The PVP solutions were mixed with 50 mg of illite and allowed to react for 15 h at 4 °C using a rotatory shaker. After the reaction, the excess PVP was removed using an Amicon Ultra-15 centrifugal filter (molecular weight cutoff 100 kDa; Merck Millipore, Billerica, MA, USA). The solutions of PVP-coated MPs were then lyophilized to a powder state for 3 d and stored at −20 °C before use. They were denoted as P-lite1, P-lite2, and P-lite3 based on the weight ratio of illite to PVP (1:5, 1:10, and 1:20). Bare illite MPs were prepared without PVP using the same method as mentioned above.

### 2.3. Characterization of P-Lite MPs

The developed illite and P-lite MPs were characterized using several techniques [22,23]. The hydrodynamic diameters and size distribution graphs were obtained using a Zetasizer (ELSZ; Otsuka, Osaka, Japan). The MP morphology associated with each amount of PVP coating was observed via SEM (JSM-6701F; JEOL, Tokyo, Japan). The interactions between illite and PVP were determined via FTIR (FT/IR-460 plus; Jasco, Tokyo, Japan) and XPS (VG Multilab 2000; Thermo Fisher Scientific, Waltham, MA, USA).

### 2.4. Stability of P-Lite MPs

The illite and P-lite MPs were freeze-dried for ease of storage until use. The MPs were dispersed again in PBS to confirm that their properties were maintained after lyophilization without the use of any cryoprotectant. The hydrodynamic diameter of the MPs was measured using a Zetasizer. Further, the long-term stability of the MPs was monitored for 2 weeks in PBS at 37 °C. The MP solutions were incubated at 37 °C, and their sizes were measured every week using a Zetasizer.

### 2.5. In Situ Antioxidant Activity of P-Lite MPs

The protective effect of antioxidant samples against the degradation of deoxyribose by hydroxyl radicals was evaluated [24]. An aqueous mixture of EDTA (0.1 mL, 0.1 mM), FeCl_3_ (0.1 mL, 0.1 mM), H_2_O_2_ (0.1 mL, 1 mM), potassium phosphate buffer (0.5 mL, 20 mM), and ascorbic acid (0.1 mL, 0.1 mM) was prepared to produce hydroxyl radicals. 2-Deoxy-D-ribose (0.1 mL, 3.75 mM) was added to the solution. Subsequently, 1 mL of illite and P-lite MPs (10 mg/mL) was added to the reaction mixtures. The reaction was allowed to occur for 1 h in a 37 °C incubator and terminated by adding 1 mL of TBA (1% *w*/*v* in 50 mM NaOH) and 1 mL of TCA (2% *w*/*v* in DI water). Degraded deoxyribose produced a pink chromogen upon heating the mixture at 85 °C for 20 min after the reaction. A microplate reader was used to measure the absorbance of the sample solutions at a wavelength of 515 nm. The antioxidant activity of the illite and P-lite MPs was calculated using this Equation (1) [25]:

(1) $$Antioxidant\;activity\;\left(\%\right)=\left[\frac{\left(\Delta A_{515}\;of\;sample-\Delta A_{515}\;of\;blank\right)}{\left(\Delta A_{515}\;of\;control-\Delta A_{515}\;of\;blank\right)}\right]\times 100$$

A control was prepared using DI water as a substitute for the sample solution, and a blank was prepared without H_2_O_2_ and the sample solution.

The antioxidant activity of the illite and P-lite1 MPs was further compared by monitoring the H_2_O_2_ reduction after the MP treatment [26]. The 2 mM H_2_O_2_ solution was prepared in a 50 mM phosphate buffer. Then, a 0.6 mL H_2_O_2_ (2 mM), 0.4 mL phosphate buffer (50 mM), and a 0.1 mL MP solution (5, 10, and 20 mg/mL) were sequentially added and mixed for 10 min using a rotatory shaker. The absorbance of the reaction mixtures was measured at a wavelength of 230 nm to determine the inhibition of H_2_O_2_.

In addition, the DPPH radical-scavenging activity of the illite and P-lite1 MPs was analyzed to determine their antioxidant potential [27]. First, 2 mg DPPH was dissolved in 10 mL methanol. A solution of equal volumes of DPPH and MP (50 mg/mL) was mixed in a 96-well plate and kept at room temperature in the dark for 30 min. The negative control was prepared by adding DI water to the DPPH solution. A decrease in the DPPH level was determined by measuring the absorbance of the mixtures at a wavelength of 515 nm, indicating the antioxidant activity of the MPs.

### 2.6. Antibacterial Activity of P-Lite MPs

The antibacterial activity of the P-lite MPs was quantitatively evaluated against *S. aureus* ATCC 6538 using a colony-counting method [28]. The bacteria were cultured in Luria–Bertani (LB) agar plates (BD Difco, Sparks, MD, USA) containing 1.5% agar at 37 °C. A single colony was inoculated into the LB broth and cultured overnight at 37 °C before use. This culture was suspended in fresh LB broth until the optical density (OD_600_) reached 0.1. The P-lite MPs (0.5 mg/mL) were subsequently added to each culture for evaluating bacterial growth inhibition during 24 h of incubation. DI water was used as a negative control. The suspensions were diluted to a factor of 10^6^ after incubation, and 100 μL of each test suspension was spread over an agar plate using glass beads. The number of colonies that appeared after overnight incubation was counted to determine the viability of *S. aureus*.

### 2.7. In Vitro Cytotoxicity and Antioxidant Activity of P-Lite MPs

The biocompatibility of P-lite1 was analyzed using NIH 3T3 fibroblasts. The cells were seeded in a 96-well plate at a density of 10,000 cells/well. They were incubated up to approximately 80% confluency in a humidified atmosphere of 5% CO_2_ at 37 °C. The P-lite1 (0.01–1.0 mg/mL) was then treated for 24 h. The control group was treated with cell media instead of a sample solution. After incubation, Cell Counting Kit-8 (CCK-8) solution (Dojindo Laboratories, Kumamoto, Japan) was used to analyze the cell viability. It produced an orange-colored formazan from viable cells that exhibit absorbance at a wavelength of 450 nm. The cell viability after the MP treatment was calculated using this Equation (2):

(2) $$Cell\;viability\;\left(\%\right)=\left(\frac{\Delta A_{450}\;of\;sample}{\Delta A_{450}\;of\;control}\right)\times 100$$

In addition, the in vitro antioxidant activity of P-lite1 was assessed by reducing the in vitro ROS level induced by the oxidative stress agent, H_2_O_2_. NIH 3T3 (10,000 cells/well) in a 96-well plate was stimulated by 100 μL H_2_O_2_ (5 μM) and subsequently treated by 100 μL P-lite1 (0–100 μg/mL) for ROS scavenging during a 4 h incubation. Each 200 μL cell media were treated in control groups to determine the natural ROS level of the cells without the MP treatment. Then, the intracellular fluorescent dye, H2DCFDA (10 μM), was used to treat the cells after washing several times with PBS. Dichlorofluorescein was produced in response to in vitro ROS during a 1 h incubation in the dark, and its fluorescence (Ex/Em = 485/535 nm) was measured using a microplate reader. The ROS level% was calculated based on the fluorescence intensity of the control and sample groups.

### 2.8. Statistical Analysis

Every experiment was repeated in triplicate, and the resulting data were averaged to be expressed as the mean ± standard deviation. Student’s *t*-test was used to compare statistically significant differences between any two experimental groups. The differences were considered to be statistically significant if *p* < 0.05, highly significant if *p* < 0.01, very highly significant if *p* < 0.001, or not statically significant if *p* > 0.05. Next, symbols were allocated to indicate statistical significance, namely # for *p* > 0.05, * for *p* < 0.05, ** for *p* < 0.01, and *** for *p* < 0.001.

## 3. Results and Discussion

### 3.1. Preparation and Characterization of P-Lite MPs

**Figure 1:**
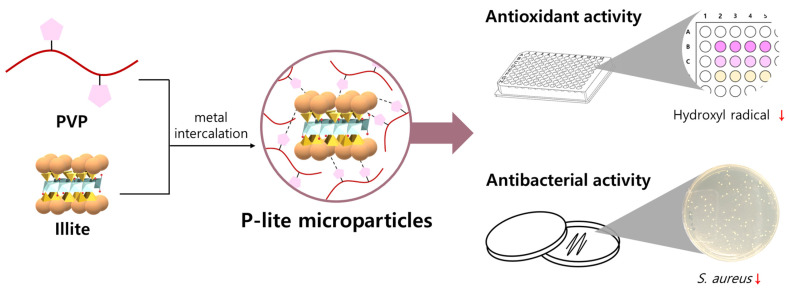
Schematic preparation of PVP-stabilized illite microparticles. PVP, polyvinylpyrrolidone; P-lite, PVP-stabilized illite; *S. aureus*, *Staphylococcus aureus*.

**Figure 2:**
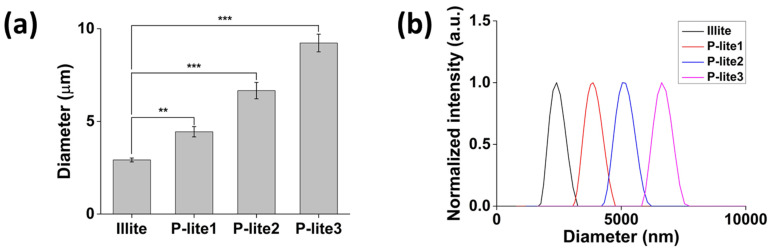
Characterization of illite and P-lite MPs. (**a**) Hydrodynamic diameters and (**b**) size distribution graphs of illite and P-lite MPs prepared using different weight ratios of PVP to illite. PVP, polyvinylpyrrolidone; P-lite MP, PVP-stabilized illite microparticles; P-lite1, 1:5 weight ratio of illite:PVP; P-lite2, 1:10 weight ratio of illite:PVP; P-lite3, 1:20 weight ratio of illite:PVP; a.u., arbitrary units; ** *p* < 0.01; *** *p* < 0.001.

**Figure 3:**
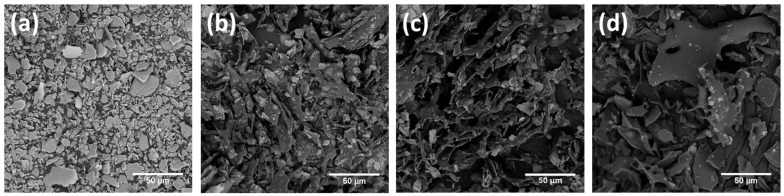
Scanning electron microscopy images of (**a**) illite, (**b**) P-lite1, (**c**) P-lite2, and (**d**) P-lite3 MPs. P-lite1, P-lite2, and P-lite3 MPs were prepared using a weight ratio of PVP to illite of 1:5, 1:10, and 1:20, respectively. PVP, polyvinylpyrrolidone; P-lite MPs, PVP-stabilized illite microparticles.

Illite MPs were successfully prepared in an aqueous solution via metal intercalation between PVP and illite to enhance the antioxidant and antibacterial activities of illite (Figure 1). Based on the weight ratio of illite to PVP, a large amount of PVP was coated on the surface of the illite. Similarly, Stuart et al. found that the adsorption of PVP increased with an increasing polymer dosage [29]. Consequently, the hydrodynamic diameters of the P-lite MPs increased from 4.4 ± 0.3 μm to 6.7 ± 0.4 μm and 9.2 ± 0.5 μm with an increase in the amount of PVP, whereas that of bare illite was 2.9 ± 0.1 μm (Figure 2a). The size distributions of the MPs indicated that these sizes were fairly uniform (Figure 2b). Two-dimensional sheets of illite and P-lite MPs were observed via SEM (Figure 3). Bare illite possessed the typical layered structure of clay minerals observed previously [30]. The SEM images of the P-lite MPs showed that the clay flakes increased in size and contained relatively smoother surfaces after polymer coating.

**Figure 4:**
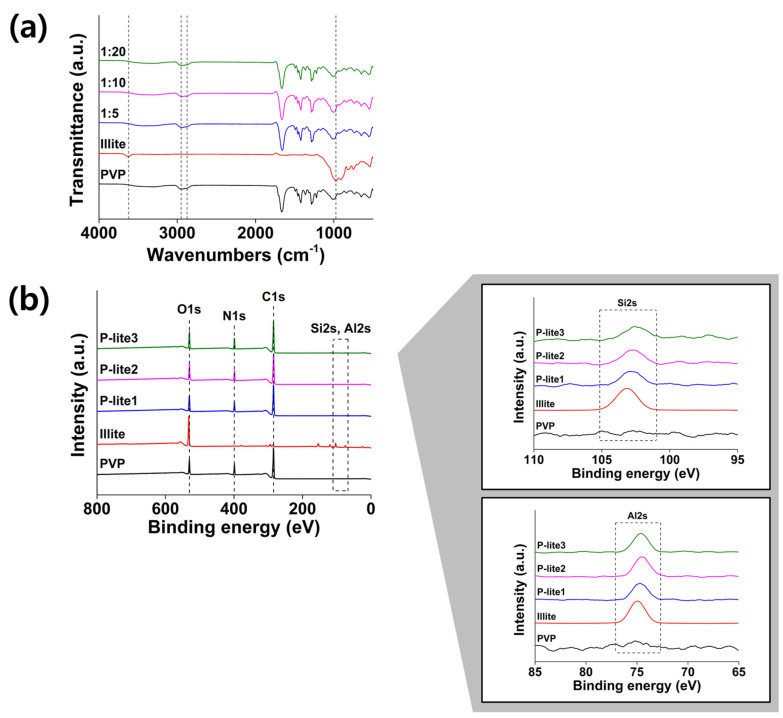
(**a**) Fourier transform infrared and (**b**) X-ray photoelectron spectra of PVP, illite, and P-lite MPs. PVP, polyvinylpyrrolidone; P-lite MP, PVP-stabilized illite microparticles; P-lite1, 1:5 weight ratio of illite:PVP; P-lite2, 1:10 weight ratio of illite:PVP; P-lite3, 1:20 weight ratio of illite:PVP; a.u., arbitrary units.

FT-IR and XPS were performed to analyze the chemical interactions between the illite and PVP in P-lite MPs. The characteristic peaks of PVP were observed in the FTIR spectra, as previously reported [31]. The peaks at 2952 cm^−1^, 1667 cm^−1^, and 1280 cm^−1^ were attributed to the C-H stretching, C=O stretching, and C-N stretching vibrations of PVP, respectively (Figure 4a) [32]. The FTIR spectra of illite showed Si-O stretching and Al-O-H bending peaks at 973 cm^−1^ and 898 cm^−1^, respectively [33]. In addition, the bands in the range of 824 cm^−1^ and 534 cm^−1^ were attributed to the Si-O stretching and bending modes in illite. After performing polymer coating, these illite-based peaks were absent in the spectra of the P-lite MPs, indicating that PVP was coated on the surface of the illite MPs. Further, the slight red-shift of the C=O stretching bands to 1659 cm^−1^ possibly indicates the formation of hydrogen bonds between the C=O of PVP and the silanols in illite [34,35]. The chemical compositions of PVP, illite, and P-lite MPs were analyzed using XPS (Figure 4b). The elements O1s, N1s, C1s, Si2s, and Al2s were present at 530, 400, 284, 103, and 75 eV in their XPS spectra, respectively [36]. The illite was found to comprise its typical elements, including O, Si, and Al. The organic components of PVP, namely C, N, and O, were found in the XPS spectra of the P-lite MPs, indicating that the PVP was coated on the surface of the illite. The Si2S region of the illite was red-shifted to 102 eV after PVP coating. This peak was assigned to Si-O-C bonds, which determine the chemical interaction between PVP and illite, referred to as metal intercalation [37]. A similar phenomenon was observed in the Al2s region of the P-lite MPs. Taken together, these findings indicate that the metal intercalation of PVP into illite was successful.

### 3.2. Stability of P-Lite MPs

**Figure 5:**
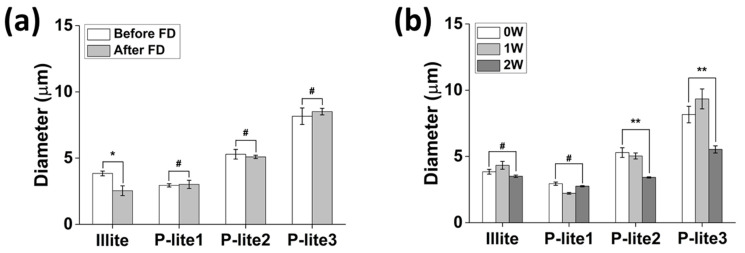
Stability analysis of illite and P-lite MPs in a biological buffer at 37 °C. (**a**) Change in diameters of illite and P-lite MPs after lyophilization and redispersion in phosphate-buffered saline. (**b**) Hydrodynamic diameters of illite and P-lite MPs after 2 weeks of storage at 37 °C. PVP, polyvinylpyrrolidone; P-lite MP, PVP-stabilized illite microparticles; P-lite1, 1:5 weight ratio of illite:PVP; P-lite2, 1:10 weight ratio of illite:PVP; P-lite3, 1:20 weight ratio of illite:PVP; FD, freeze-drying; 0W, 0 weeks; 1W, 1 week; 2W, 2 weeks; # *p* > 0.05; * *p* < 0.05; ** *p* < 0.01.

Lyophilization is performed to dry the MPs in a solution to facilitate transportation and storage until use. This process may exert considerable stress on the MPs, causing an increase in their size and aggregation. Thus, lyophilization stability is often achieved by using lyoprotectants, such as sucrose and trehalose [38]. In this study, the freeze-dried MPs were readily dispersed in a biological buffer without using any lyoprotectant. The stability of the illite and P-lite MPs was analyzed by monitoring the changes in their hydrodynamic diameters after lyophilization and after two weeks of storage in PBS (Figure 5). Their hydrodynamic diameters were maintained before and after freeze-drying (Figure 5a). The diameters of P-lite1, P-lite2, and P-lite3 MP were 2.9 ± 0.1 μm, 5.3 ± 0.4 μm, and 8.2 ± 0.6 μm before lyophilization. They barely changed after lyophilization (3.0 ± 0.3 μm, 5.1 ± 0.1 μm, and 8.5 ± 0.2 μm, respectively). However, the diameter of bare illite changed after lyophilization from 3.8 ± 0.2 μm to 2.5 ± 0.4 μm with a statistical difference (*p* < 0.05), which indicates that P-lite MPs can be lyophilized for long-term storage prior to their future applications.

The illite and P-lite1 MPs were stable during two weeks of storage in a biological buffer without any change in their hydrodynamic diameters (Figure 5b). Their diameters after stability analysis were 3.5 ± 0.1 μm and 2.8 ± 0.1 μm. The P-lite1 MPs were suspended for a relatively longer period in the biological buffer than the bare illite MPs because the hydrophilic PVP provides steric and electrostatic stability due to its amide and methylene groups [39]. In contrast, the hydrodynamic diameters of the P-lite2 and P-lite3 MPs decreased, respectively, to 3.4 ± 0.1 μm and 5.5 ± 0.3 μm after two weeks of storage in the buffer. The PVP might have desorbed from the surface of the illite due to its weak interaction.

### 3.3. Antioxidant Activity of P-Lite MPs

**Figure 6:**
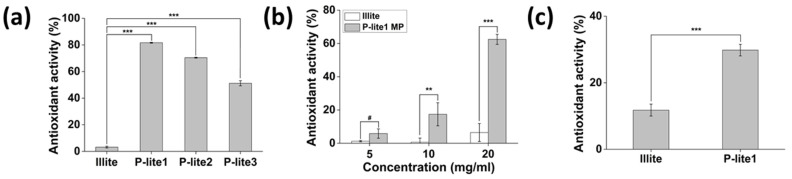
Antioxidant activity of illite and P-lite MPs. The antioxidant activity was evaluated via (**a**) hydroxyl radical-scavenging assay, (**b**) H_2_O_2_ radical-scavenging assay, and (**c**) DPPH radical-scavenging assay. P-lite MP, Polyvinylpyrrolidone-stabilized illite microparticles; P-lite1, 1:5 weight ratio of illite:polyvinylpyrrolidone; P-lite2, 1:10 weight ratio of illite:polyvinylpyrrolidone; P-lite3, 1:20 weight ratio of illite:polyvinylpyrrolidone; # *p* > 0.05; ** *p* < 0.01; *** *p* < 0.001.

The free radical-scavenging activity of illite and P-lite MPs was measured to evaluate their antioxidant activity (Figure 6). Bare illite is a poor antioxidant material and was found to scavenge only 3.2 ± 0.7% of the hydroxyl radicals (Figure 6a). Notably, the P-lite MPs exhibited high radical-scavenging activity (*p* < 0.001). Considering that PVP does not show any antioxidant activity, the radical-scavenging activity of the P-lite MPs may have increased because the PVP coating on the illite surface enhances the interaction of MPs with hydroxyl radicals in an aqueous solution [40]. In addition, the radical-scavenging activity of the relatively small P-lite MPs may be high because of their larger surface area compared to large MPs for facilitating interaction with radicals. This is supported by our results showing that the P-lite1 MP exhibited the highest hydroxyl radical-scavenging activity of 81.7 ± 0.4%. The antioxidant activities of P-lite2 and P-lite3 MPs were 70.4 ± 0.3% and 51.2 ± 1.9%.

The P-lite1 MP was also effective in inhibiting H_2_O_2_ (Figure 6b). Its antioxidant activity increased dose-dependently from 5.8 ± 2.8% and 17.4 ± 6.9% to 62.5 ± 3.0%, while the illite could barely scavenge H_2_O_2_ (6.4 ± 5.4% at 20 mg/mL). Similarly, the DPPH radical-scavenging activity of illite alone was very low (Figure 6c). However, that of P-lite1 MP was enhanced to 29.8 ± 1.7% with a statistically significant difference (*p* < 0.001). This result correlates with Jeong et al., who found that illite shows low DPPH radical-scavenging activity because of its low cationic-exchange capacity [41]. However, the P-lite1 MP exhibited antioxidant activity three times higher than illite alone, suggesting that PVP coating on illite surfaces offers more chances to interact with radicals for their reduction. Therefore, an illite to PVP weight ratio of 1:5 may considerably increase the radical-scavenging activity of illite.

### 3.4. Antibacterial Activity of P-Lite MPs

**Figure 7:**
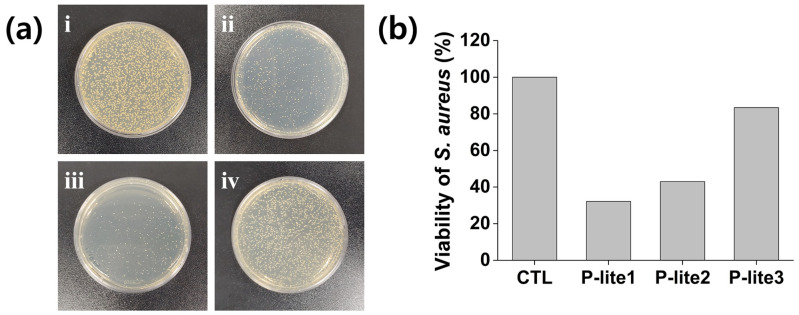
Antibacterial activity of P-lite MPs. (**a**) Photographs of *Staphylococcus aureus* colonies (**i**) without (CTL) and with treatment with (**ii**) P-lite1, (**iii**) P-lite2, and (**iv**) P-lite3 MPs. (**b**) Viability of *S. aureus* after treatment with P-lite MPs. CTL, control; P-lite MP, Polyvinylpyrrolidone-stabilized illite microparticles; P-lite1, 1:5 weight ratio of illite:polyvinylpyrrolidone; P-lite2, 1:10 weight ratio of illite:polyvinylpyrrolidone; P-lite3, 1:20 weight ratio of illite:polyvinylpyrrolidone.

Illite has been previously reported to show antibacterial activity; however, it did not inhibit *S. aureus* growth in our study due to its poor solubility in aqueous solutions [42]. The P-lite MPs inhibited the growth of *S. aureus* to a great extent compared to that obtained using the control (Figure 7). The viability of *S. aureus* decreased to 32% after treatment with the P-lite1 MPs. Notably, the increase in the amount of PVP coating on the illite from P-lite1 to P-lite3 decreased the antimicrobial activity, indicating that the relatively small P-lite MPs could show high antibacterial activity against *S. aureus*. This is inconsistent with the result of D. Bhatia et al., who determined a good antibacterial activity of PVP against *S. aureus* [43]. The decrease in antibacterial activity with an increase in PVP coating may be attributed to a decrease in the interaction and bioavailability of bactericidal metal elements in bacteria with an increase in PVP coating, similar to the results of the hydroxyl radical-scavenging activity assays [44], indicating the synergistic antibacterial activity of P-lite MPs. Therefore, an illite to PVP weight ratio of 1:5 may considerably inhibit *S. aureus* growth via illite MP treatment.

### 3.5. In Vitro Cytotoxicity and Antioxidant Activity of P-Lite MPs

**Figure 8:**
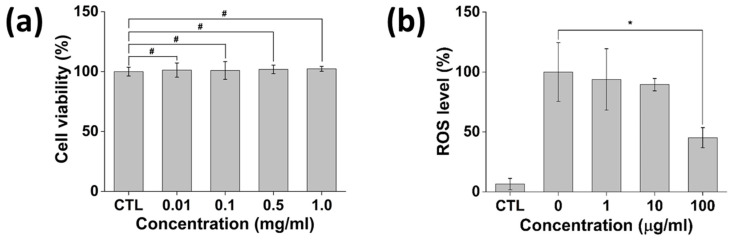
In vitro assays of P-lite1 MPs. (**a**) Cytotoxicity of P-lite1 MPs. (**b**) In vitro ROS scavenging activity of P-lite1 MPs. CTL, control; ROS, reactive oxygen species; # *p* > 0.05; * *p* < 0.05.

The cytotoxicity of the P-lite1 MP was analyzed by monitoring the change in cell viability after MP treatment in NIH 3T3 fibroblast cells (Figure 8a). It was found that more than 95% of cells survived after treatment with 0–1 mg/mL P-lite1 MPs (100.0 ± 3.7%, 101.3 ± 5.9%, 101.0 ± 7.4%, 101.8 ± 3.5%, and 102.3 ± 2.0%, respectively). Notably, the highest concentration of P-lite1 MPs, 1 mg/mL, did not show any cytotoxic effect, exhibiting cell viability of 102.3 ± 2.0%. This result correlates with that of Seong et al., who determined the biocompatibility of illite and illite-polyethylene composite [45]. Further, PVP is one of the safe materials for biomedical use approved by the U.S. Food and Drug Administration, which implies that the P-lite1 MP possesses an in vitro biocompatibility for its biomedical application [46].

The antioxidant potential of P-lite1 MPs in various biomedical fields was determined in vitro using NIH 3T3 fibroblasts (Figure 8b). The cells with and without H_2_O_2_ treatment exhibited the highest and lowest ROS levels, respectively. H_2_O_2_-induced ROS accumulation was resisted by the P-lite1 MP. Accordingly, the ROS level decreased after an increasing amount of the P-lite1 MP was treated. Notably, the P-lite1 MP at 100 μg/mL was able to scavenge 55% of the ROS (*p* < 0.05). The mechanism underlying the ROS regulation of clay and polymer composite still requires further investigation. However, several studies have demonstrated the effect of clay minerals and derived composites related to in vitro oxidative stress [47]. While there remains an unmet need to counteract toxic ROS, P-lite1 MPs are a potent antioxidant material with a strong potential for future use.

## 4. Conclusions

Illite was stabilized by coating the surface of PVP to enhance its bioavailability and antioxidant and antibacterial activities. An increasing amount of PVP was coated on a fixed amount of illite, resulting in a size increase of the P-lite MPs. The illite plates were observed to have a typical layered structure before and after PVP coating. Further, it was determined that PVP was chemically bound on the surface of illite based on FTIR and XPS analyses. The developed P-lite MPs maintained their hydrodynamic diameters after lyophilization and after 2 weeks of storage in a biological buffer at 37 °C, indicating good stability after coating with PVP. Notably, the P-lite MPs possessed high antioxidant and antibacterial activities after coating PVP on the surface of illite. In addition, the MPs showed effective free radical-scavenging activity in vitro without any cytotoxicity. Therefore, P-lite MPs may be used as antioxidants and bactericides in biomedical applications.

## Data Availability

Not applicable.
